# Foam-Based Electrophoretic Separation of Charged Dyes

**DOI:** 10.1021/acs.langmuir.2c02228

**Published:** 2022-11-02

**Authors:** Matthieu Fauvel, Anna Trybala, Dmitri Tseluiko, Victor Mikhilovich Starov, Himiyage Chaminda Hemaka Bandulasena

**Affiliations:** † Department of Chemical Engineering, Loughborough University, Loughborough, Leicestershire LE11 3TU, U.K.; ‡ Department of Mathematics, Loughborough University, Loughborough, Leicestershire LE11 3TU, U.K.

## Abstract

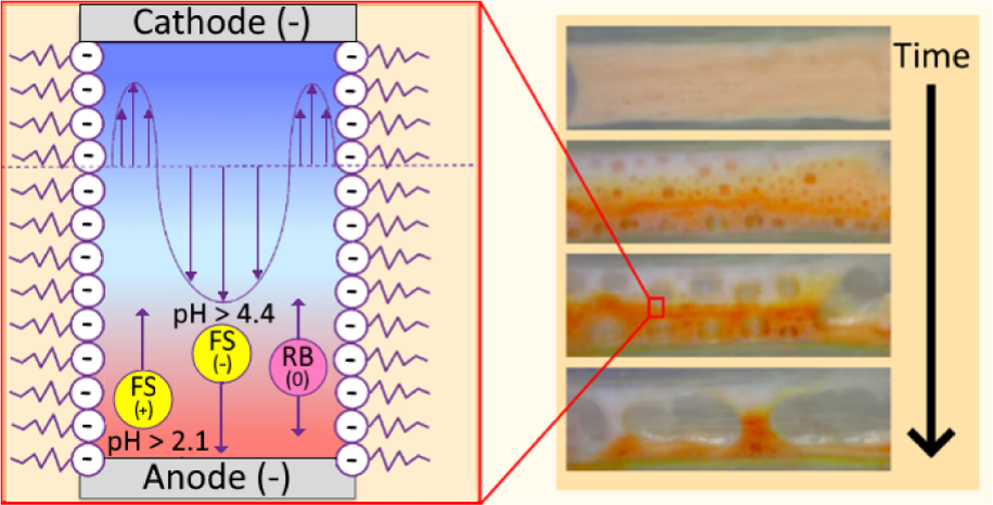

Electrophoretic separation of a fluorescent dye mixture, containing rhodamine B (RB) and fluorescein, in liquid foams stabilized by anionic, cationic, or non-ionic surfactants in water–glycerol mixtures was studied in a custom-designed foam separation device. The effects of the external electric field applied across the foam and the initial pH of the solution on the effectiveness of separation were also studied. The fluid motion due to electroosmosis and the resulting back pressure within the foam and local pH changes were found to be complex and affected the separation. Fluorescein dye molecules, which have a positive or negative charge depending on the solution pH, aggregated in the vicinity of an electrode, leaving a pure band of neutral dye RB. The effectiveness of the separation was quantified by the percentage width of the pure RB band, which was found to be between 29 and 42%. This study demonstrates the potential of liquid foam as a platform for electrophoretic separation.

## Introduction

Electrokinetic phenomena have found many applications since their discovery almost 200 years ago.^1^ A common method is electrophoresis, used in biochemistry to separate complex biological samples into their constituent components, whether it be individual proteins^2^ or entire cells^2^ based on their mobility under an applied electric field. Common electrophoresis techniques, such as gel or capillary electrophoresis, typically rely on either suspending samples in porous materials or in fixed solid-wall capillaries. Liquid foams are composed of gas bubbles suspended in a continuous liquid medium. The lamellae, plateau borders, and nodes of liquid foams effectively provide a network of deformable nano-/micro-channels bound by the gas–liquid interfaces instead of solid walls. Liquid foams could provide a novel platform for electrophoretic separation operations, providing a cheap and flexible alternative without the need for precisely fabricated solid channels. Thin films of liquid between foam bubbles can have thicknesses in the order of 10 nm,^3^ meaning liquid foams can act as a network of deformable nanochannels. Unique separation methods may be developed that take advantage of the coupling of physical forces present in such nano-scale systems as polarization effects and steric interactions affect the complex motion of macromolecules.^4^ Novel separation methods that take advantage of the high surface area inside liquid foams are desired as unique analyte dynamics arise in micro- and nano-scale channels^5−8^ or use nanochannels as a filtration mechanism for electrophoretic separations.^9^

Liquid foams are stabilized by surfactants, which adsorb onto the gas–liquid interfaces. The choice of surfactant used affects the interface properties, especially the surface tension and the interfacial charge. The surface charge attracts oppositely charged counterions from the bulk liquid, resulting in a layer of ions in the proximity of the interface, known at the electrical double layer (EDL).^10^ Applying an electric field across charged surfactant-laden interfaces induces electroosmotic flow (EOF) as ions in the EDL are dragged by the electric field, establishing a fluid flow.^10^ EOF will also affect the motion of analytes in an electrophoretic separation system and depending on its strength and direction relative to the electrophoretic mobility of each analyte, it may help or hinder the separation.^11,12^ It is important to consider the effects of EOFs generated in liquid foams for different surfactant types on potential electrophoretic separations as well as on the stability and lifetime of the foam. The effect of EOF on freely suspended surfactant-laden films has been investigated by several researchers,^13−15^ and the effects on the stability of liquid foams have been previously investigated.^15,16^ While electrophoresis in a free liquid film has been investigated by,^17^ separation of charged molecules inside a three-dimensional liquid foam has not been demonstrated. In our previous work, it was shown that the foam stability depends on the surfactant type and the strength of the electric field applied;^16^ therefore, it is interesting to know whether the required separation can be achieved before the foam starts to collapse.

This paper presents a custom-made foam separation device and demonstrates its operation using a dye mixture containing charged/uncharged dyes, aiming to separate them based on their differing electrical charge, electrophoretic mobility, and interaction with the gas–liquid interface. The effects of surfactant type, starting pH, and the applied electric field strength on the separation were investigated, with a view to optimize the effectiveness of separation.

## Materials and Methods

### Solution Preparation

Solutions were prepared by mixing 25 g of Milli-Q water (15 MΩ·cm deionized water) with 25 g of glycerol. Glycerol was added to the solution to increase the viscosity and reduce foam drainage. 100 μL of 1 M phosphate buffer solution (Sigma-Aldrich, UK) was added to the solution to regulate the initial pH to 7, adjusting the bulk molarity to 2 mM. A dye mixture containing 9.45 mg of sodium fluorescein and 2.31 mg of RB was added to the solution, with respective concentrations of 0.05 mM and 0.1 mM. At pH 7.2, RB is neutral, while fluorescein has a charge of −2.^18^ From this solution, four test solutions were prepared, labeled 1–4. Anionic surfactant sodium dodecyl sulfate (SDS) was added to solution 1 at a 1 critical micelle concentration (CMC). Cationic surfactant myristyltrimethylammonium bromide (MTAB) was added to solution 2 at 1 CMC. Non-ionic surfactant Triton X-100 was added to solution 3 at 1 CMC. Both SDS and Trition X-100 were added to solution 4, each at 1 CMC. Two more solutions, numbered 5 and 6, were prepared identical to solution 4, but with 1 mL of 1 M phthalate buffer added to solution 5 and 1 mL of 1 M borate buffer added to solution 6 in place of phosphate buffer. Chemical structures of the surfactants used in this study are presented in Supporting Information: Figure S1. The viscosity of all the solutions is 4.01 × 10^–3^ Pa·s. CMCs of all surfactants are presented in Table 1.

**Table 1:** List of Surfactants Used in the Experiments, Their Type, and CMC

name	surfactant type	CMC
SDS	anionic	8.2 mM^19^
MTAB	cationic	4–5 mM^20^
Triton X-100	non-ionic	0.24 mM^21^

### Experimental Device

**Figure 1:**
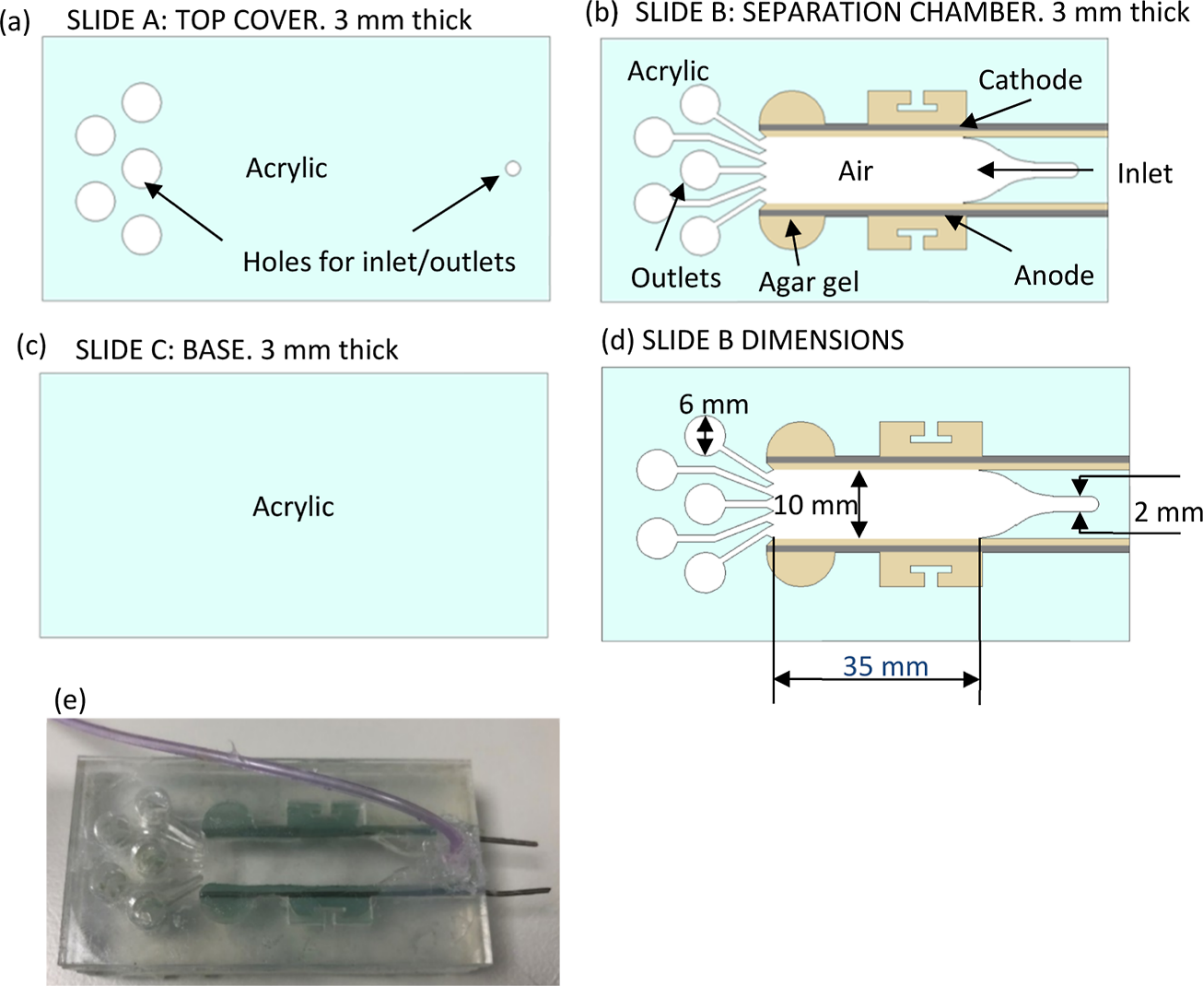
Foam separation cell; (a) top cover slide, (b) separation chamber slide, (c) base slide, (d) slide B dimensions, and (e) photograph of the assembled device.

A foam separation cell was constructed by cutting a series of slides out of 3 mm-thick acrylic sheets. Acrylic was chosen as the device material based on investigation conducted in ref (16). Once assembled, the slides were layered together to form the separation device. The layout of each slide is shown in Figure 1, showing each slide in order from top to bottom.

The assembly process was as follows: slide B was sealed to the top of slide C using neutral cure silicone sealant. Electrodes used were 1 mm diameter platinized titanium rods. In order to prevent bubble formation at the electrodes due to electrochemical effects, the electrodes were coated with agarose gel. For this coating, 20 mL of deionized water was mixed with 2 mL of 1 M phosphate buffer and 180 mg of agarose. The agar solution was heated to 150 °C for 40 min and poured into the device until the air chamber in slide B is filled. The device was then placed in a fridge and allowed to cool for 1 h. Once cooled, the gel was cut to form the separation chamber between the electrodes, ensuring that both electrodes remain covered by the agar gel. The gel prevents gaseous byproducts from entering the foam, and the buffer ensures the gel starts at neutral pH. Once the agar gel was properly cast, slide A was sealed over the top of slide B using a neutral cure silicone and allowed to set. Five outlet channels were included at various width-wise positions across the cell to facilitate recovery of separated dyes. However, this study was carried out in batch mode, and the outlets were not used. In continuous operation, foam will continuously flow through the separation chamber and will be collected via the five outlets, which will contain separated fractions from the mixture.

The operation of the device is as follows: foam was generated by using the double syringe method,^22^ where one syringe was filled with 0.5 mL of foaming solution and the other filled with 1.5 mL of air. The two syringes were connected through a tube, and fluid was passed between the syringes for 2 min to generate the foam. The bubble sizes generated through this method are presented in Table 2. Once the foam was generated, it was promptly injected into the device inlet shown in Figure 1b until the separation chamber was fully filled. Immediately after filling with the foam, the electrodes were attached to a DC signal generator (Thurlby Thandar PL30QMD), and an electric field of 1000 V/m was applied across the foam. The device was placed under a camera (Logitech C270) and time lapse of the separation was recorded. This process was repeated for solutions 1, 2, and 3, which contain SDS, MTAB, and Triton X-100, respectively, with an electric field strength of 1000 V/m. Next, the electric field strength was varied between 500 and 2000 V/m for foam made of solution 4 (containing both SDS and Triton X-100 at pH 7). Finally, foams prepared with a surfactant mixture of SDS and Triton X-100 with phthalate (solution 5, initial pH = 4) and borate (solution 6, initial pH = 10) buffers in place of phosphate buffer were tested at 1000 V/m. All experiments were repeated 3 times.

**Table 2:** Foam Bubble Sizes (in μm) for All the Test Solutions Immediately after Generation Using the Double Syringe Method

surfactant	mean	min	max	standard deviation
SDS	11.5	3.7	29.4	4.38
MTAB	11.4	3.9	30.8	4.24
Triton X-100	11.5	3.7	30.1	4.40
SDS/Triton X-100	11.7	3.9	30.9	4.25

## Results and Discussion

Both fluorescent dyes used are pH-sensitive. The fluorescence intensity and color of RB drops as pH increases, changing from bright pink color in acidic conditions to colorless in basic conditions.^23−25^ Despite the change in visibility, the charge of RB remains neutral. Fluorescein exists in multiple forms, depending on the solution pH: dianonic at pH > ∼6.7, monoanionic at pH > 4.3, neutral at 2.1 < pH < 4.3, and cationic below pH 2.1.^18,26^ The fluorescence of fluorescein dye increases with pH, ranging from bright yellow/green at high pH to a darker orange color at low pH.^18,27^ When an electric field is applied across the device, the electrochemical reactions that occur at the electrodes will cause a pH gradient to form. The pH changes in the foam are visualized using a universal indicator during preliminary stages and shown in Supporting Information: Figure S2. The pH changes observed agree well with the previous experiments and simulations.^17,28^

In foams the overall gas–liquid interfacial area per unit volume is very high, which in turn can accommodate a significant amount of surfactant molecules in the adsorption layer, giving rise to depletion of surfactants in the bulk phase. Therefore, it is important to estimate the concentration of surfactants adsorbed to interfaces and the surface excess concentration for this system. According to Sachin et al.,^29^ surface excess concentration for SDS-rich water is estimated to be 3.36 μmol/m^2^ using surface tension measurements and Gibb’s isotherm. Since the interfacial concentration is much higher than the concentration of a hypothetical surface in the liquid bulk, surface excess concentration can be approximated with the interfacial concentration. The interfacial area of the foam is calculated as ∼0.41 m^2^ for this device using foam bubble size measurements. Therefore, surfactant molecules needed to saturate the interface are estimated to be 1.17 × 10^12^. Based on the experimental data, (SDS concentration in the test solution and liquid volume used to generate the foam) 1.92 × 10^12^ SDS molecules are present is the system. These estimates confirm that all interfaces are saturated and excess SDS molecules are available in the system. Calculations for MTAB are similar (based on DTAB data^29^), that is, interfaces are fully saturated with surfactants, and there are excess MTAB molecules in the system.

### Effect of the Surfactant Charge on Separation

**Figure 2:**
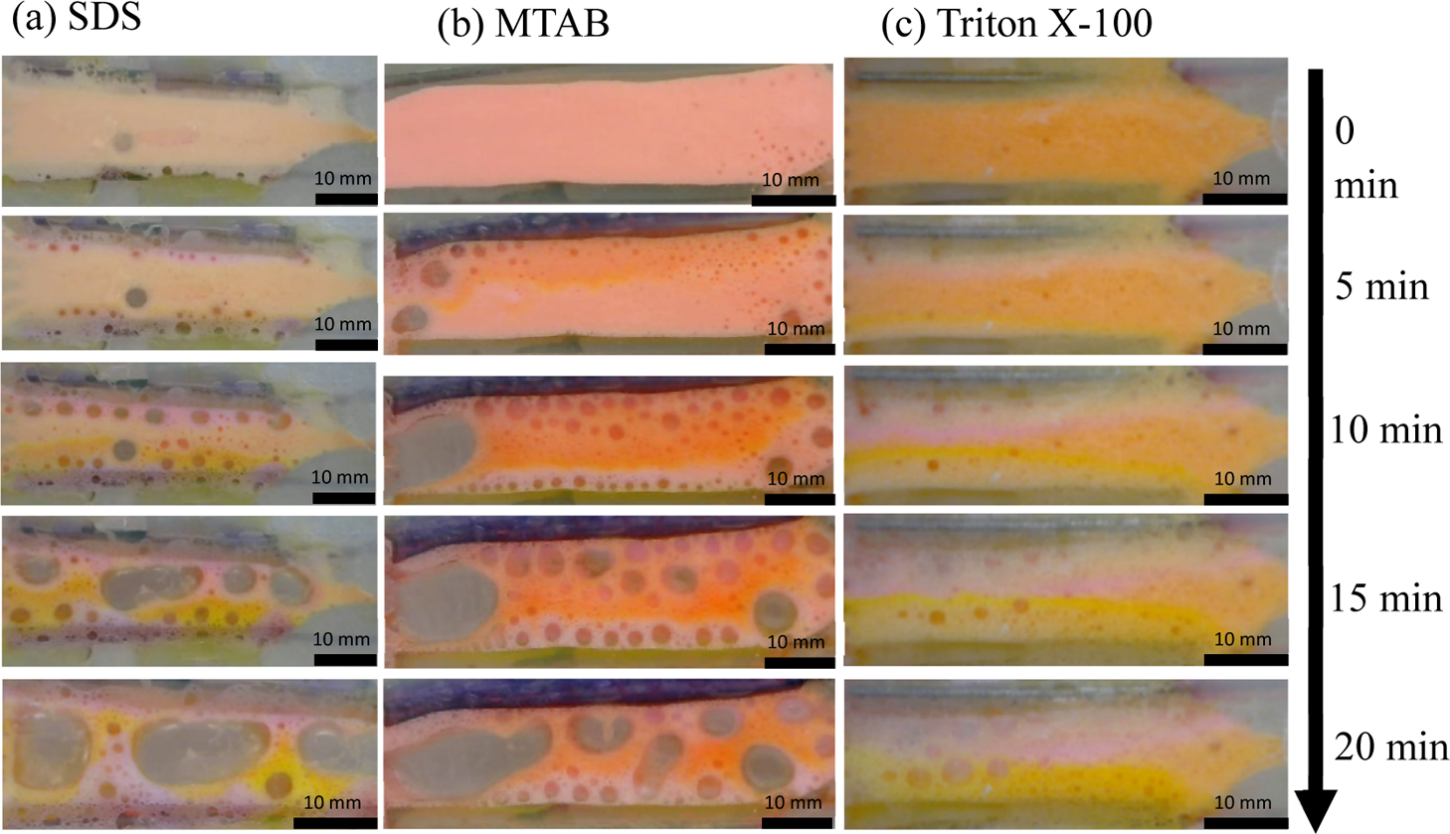
Time lapse images for (a) SDS, (b) MTAB, and (c) Triton X-100 at 1000 V/m and pH 7. The cathode is situated at the top of each image and the anode at the bottom. All experimental repeats showed similar separation behavior.

To study the effect of the surfactant charge (hence, the charge of the EDL) on separation, experiments were done using solutions 1, 2, and 3. Images from these experiments are shown in Figure 2.

For all surfactants, the foam containing the dye mixture starts out as one solid orange color (slightly different shades for each case) as the RB (pink) and fluorescein (yellow) are well mixed (pH = 7). Foams showing the color of both pure RB and pure fluorescein for all surfactant mixtures are shown in the Supporting Information, Figure S6 for comparison. For all cases, the application of an electric field results in a visible separation or concentration of the dyes. The effectiveness of the separation or concentration of a dye can be quantified by the percentage width of the color bands formed. At the point at which the separation is evaluated (*t*_s_), most of the foam should be intact and the already separated bands should remain unmixed. Pure RB bands of 29% (*t*_s_ = 10 min), 36% (*t*_s_ = 15 min), and 42% (*t*_s_ = 10 min) were observed near the cathode for solutions 1, 2, and 3, respectively. These percentage widths were calculated as widths representing the average along the separation chamber, using five equidistant points from the inlet to the outlet.

**Figure 3:**
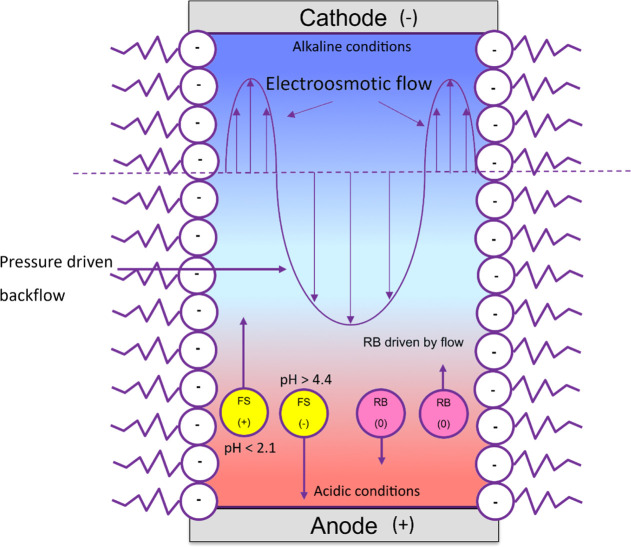
Schematic of forces acting on FS and RB molecules inside foam generated by anionic surfactant SDS under an external electric field.

When an external electric field is applied to a foam, three main effects can be expected. First, a pH gradient starts to form as electrochemical reactions occur at the electrodes.^17^ This will lead to acidic conditions at the anode (bottom electrode), and alkaline conditions at the cathode (top electrode). Second, a complex flow pattern will be established within the liquid phase. For SDS foam, the air–liquid interface is negatively charged by the adsorption of anionic surfactants; hence, positive counterions are attracted to this interface, establishing an EDL. On the application of an external electric field, these positive ions are attracted toward the cathode, creating a flow in the fluid adjacent to the EDL, establishing EOF toward the cathode, as shown in Figure 3. Since the average foam bubble diameter (11 μm) is much smaller than the channel depth (3 mm), the surface area of the liquid–acrylic interface can be assumed to be negligible compared to the gas–liquid interface of the 3D foam, and thus the EOF at gas–liquid interfaces will dominate the flow characteristics within the foam. As the cell is closed, a backflow is developed in the opposite direction to the EOF away from the gas–liquid interfaces to maintain continuity as shown in Figure 3, also reported by ref.^30^ Third, charged fluorescein dye molecules will interact with the charged gas–liquid interface and undergo electrophoresis. Initially, the fluorescein is negatively charged (at pH = 7) and so will migrate toward the anode. The negatively charged fluorescein molecules will be repelled by the negatively charged gas–liquid interfaces; therefore, fluorescein dye will typically be situated away from the gas–liquid interfaces, toward the middle of the foam films, plateau borders, and nodes. In these regions, pressure-driven backflow toward the anode is dominant, enhancing electrophoretic migration toward the anode. When the fluorescein dye approaches the anode, it encounters the acidic front generated by the electrochemical reactions. Depending on the pH near this electrode, the dye can change to its neutral form, in which case the dye will not electrophorese but get transported by the fluid flow or change to positive form, in which case it will undergo electrophoresis toward the cathode. If fluorescein becomes positive near the anode, it will get attracted toward the negatively charged interface and its transport toward the cathode will be enhanced by EOF. However, our preliminary experiments with a pH indicator confirmed that pH near the anode does not drop below 2 (see Supporting Information, Figure S5); therefore, fluorescein is unlikely to become positive in our experiments. This scenario is presented in the schematic shown in Figure 3.

The transport and accumulation of RB within the device is different to that of fluorescein. As RB is neutral, it is not affected by electrophoresis. However, the dye gets transported by the fluid flow within foam, and a uniform concentration of RB is present throughout the foam at any time, except near the gel where it may get absorbed. As fluorescein experiences a net movement toward the anode and transport away from the cathode region, a separation starts to develop, which is similar to the separation front reported by.^17^ At ∼5 min, a visible pink region starts to form at the cathode, where only RB is present, while a visible yellow band starts to form at the anode, where fluorescein has concentrated in the presence of RB. During the 5–10 min interval, these bands expand, and the colors becomes distinct, as more fluorescein is removed from this region. However, as this separation progresses, acidic conditions developed at the anode will lead to fluorescein charge becoming neutral. At this stage, fluorescein is transported back toward the cathode by EOF and electrophoresis until the dye reaches a region where pH > 4.4. The acidic and the alkaline fronts developed at the electrodes meet in the mid-section of the device due to fluid flow. At the optimum separation time, fluorescein is concentrated slightly away from the anode but remains very close to it, that is, concentrated in the bottom half of the channel but well away from cathode.

It is also observed that a narrow white region appears in the foam at both electrodes as RB gets absorbed into the agar gel, removing it from the foam, becoming visible as a dark pink band in the gel. The foam has started to collapse at this stage and the effects are visible. At 15 min, clear pink and yellow regions are still visible at places but mark the beginning of mixing of the separated bands. The fluorescein continues to concentrate toward the center of the device, while rhodamine is still present everywhere. The white bands at the electrodes enlarge as the dye gets absorbed into the gel. The foam collapse causes the dye bands to mix at several places. At around 20 min, the foam has collapsed further, and the separated bands have mixed considerably. Fluorescein appears to have continued moving toward the cathode in places as the acidic front of the pH gradient travels away from the anode, possibly assisted by the collapsing foam. This behavior is consistent with the mobility of fluorescein and RB previously analyzed in a free liquid film.^17^ Despite foam collapse at latter stages, distinct separations are visible around ∼10 min, and the foam containing separated bands should be withdrawn separately via the device outlets prior to foam collapse and mixing.

Figure 2b shows the time lapse images of dye separation with the MTAB-stabilized foam. After 5 min, separation becomes clearly visible, with a pink rhodamine band formed near the anode and a darker orange band forming at the cathode to the center. This is opposite to the color separation seen in the SDS case. As MTAB is cationic, the air–liquid interfaces are positively charged, and the diffusive part of the EDL is predominantly composed of negatively charged ions. This leads to the development of EOF toward the anode, which is opposite to the direction in the SDS case. The magnitude of zeta potential of the air–liquid interface stabilized with MTAB is approximately 20 mV^31^ compared to −70 mV for SDS;^32^ therefore, the magnitude of EOF and resulting backflow are expected to be lower compared to that in the SDS foam. Fluorescein is initially anionic; therefore, it will be subjected to electrophoresis toward the anode. However, a large fraction of fluorescein present in the plateau borders will be transported toward the cathode by the pressure-driven backflow. pH gradient setup by the electrochemical reactions will also aid fluorescein movement toward the cathode, that is, fluorescein reaching the anode by electrophoresis will turn neutral due to acidic conditions and will be carried away with the backflow. As a result, fluorescein concentrates away from the anode in this case as shown in Figure 2b. The mixing of fluids between the electrodes makes it difficult to predict any local pH changes (away from the electrodes) and the resulting local gas–liquid zeta potentials, which could lead to changes in the magnitude and the direction of the EOF setup initially. It is also observed that a small proportion of fluorescein dye closer to the agar gel boundary gets absorbed into it, removing fluorescein from the foam. Contrary to the SDS case, RB does not appear to visibly absorb into the gel at the anode. Foam breakdown is less drastic for MTAB foam, so the color bands do not appear to mix as much as in the SDS case.^16^ also demonstrated that MTAB-stabilized foams had improved stability under external electric fields.

The separation experiment with non-ionic surfactant, Triton X-100, is shown in Figure 2c. Some difficulty was encountered initially during this experiment with regards to successfully filling the device with foam, as the foam collapsed upon injection or simply failed to form properly, requiring multiple attempts to set up the experiment. Once injected into the device, the foam was found to be stable. The stability of non-ionic surfactant Triton X-100 is known to be minimally affected by external electric fields.^16^ As the surfactant is non-ionic, the interfacial charge is low relative to that of ionic surfactants (zeta potential of −6 mV^33^), and EOF is greatly reduced. In this case, dye movement is primarily caused by electrophoresis. After 5 min of applying the electric field, a light pink front is observed next to the cathode, similar to the SDS case, and a pale-yellow band is seen next to the anode as the acidic front grows away from the anode, changing the point where fluorescein electrophoresis stops. Little foam collapse is seen at this stage, in contrast to the SDS case. At 10 min, clear pink and yellow bands have formed, with the yellow fluorescein band accumulating from the center to the anode, with the color intensity increased toward the center of the device, with a pink rhodamine band just above it in the direction of the cathode, which gradually loses color, moving to the cathode as rhodamine visibility drops due to the alkaline pH induced at the cathode. No pink or white region is observed next to the anode, contrary to the SDS and MTAB cases, suggesting less fluorescein is being absorbed into the agar, as EOF is greatly reduced, meaning less fluorescein actually reaches the anode. Foam collapse is visible, but minimal at this stage. At 15 min, the yellow fluorescein band appears narrower and brighter, and the pink band is seemingly unchanged. Finally at 20 min, the bands have widened as foam collapse continues, and the pH gradient becomes uneven, widening the isoelectric band for the fluorescein and causing rhodamine visibility to increase again. To perform a practical separation, the Triton X-100 stabilized foam appears ideal, providing clear narrow bands after 10 min and maintaining clear separation even after foam collapse has become significant. The main drawback in the Triton X-100 case was simply the lack of initial foamability, which may potentially be resolved with appropriate rheology modifiers such as Aculyn-33.^34^

### Effect of the External Electric Field Strength on Separation

**Figure 4:**
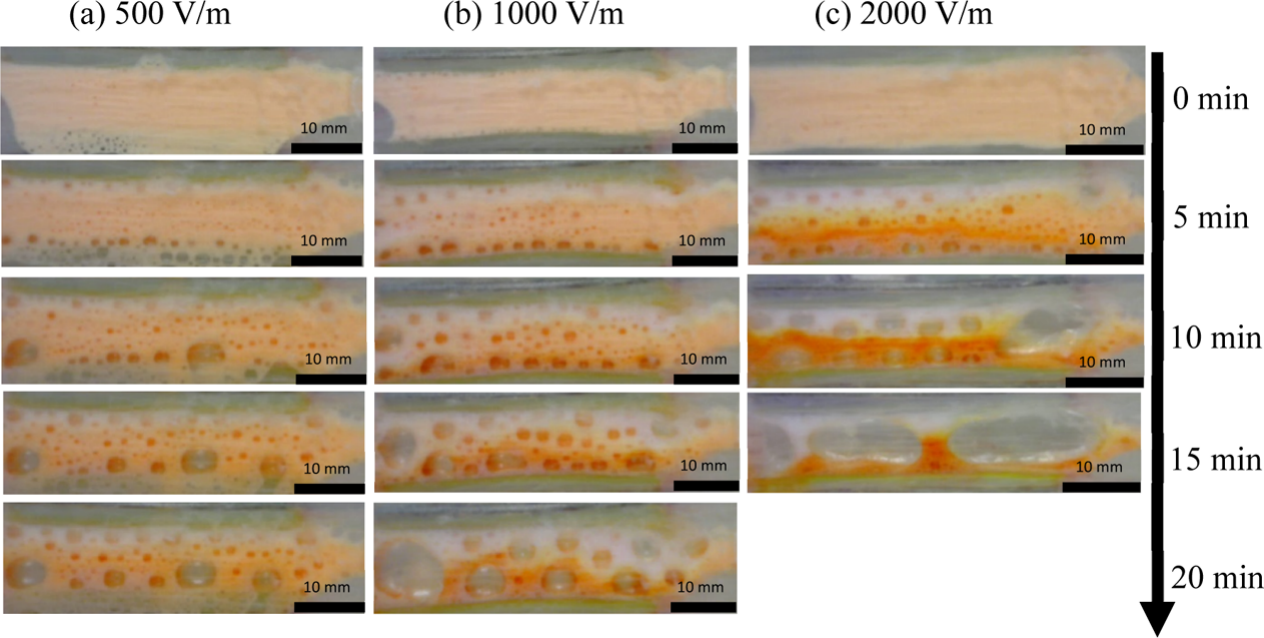
Time lapse images for SDS/Triton X-100 blend (solution 4) at pH 7: (a) 500, (b) 1000, and (c) 2000 V/m. The cathode is situated at the top edge of each image and the anode at the bottom. All experimental repeats showed similar separation behavior.

To study the effects of varying electric field strength on the separation system, another series of experiments were conducted. The results presented in Figure 2 show that foam produced with Triton X-100 remained stable for longer under an external electric field and a clear separation can be achieved compared to other surfactant cases. However, there were some issues with initial foamability using Triton X-100. According to literature, it is known that mixtures of ionic and non-ionic surfactants may attain greater stability than their individual components.^35^ To solve this foamability issue, both SDS and Triton X-100 were added to the foaming solution, labeled as solution 4. Both surfactants were added at their CMC. This provided better foamability while still retaining favorable stability under external electric fields. To compare the new foaming solution to the previous cases, the first experiment was run at 1000 V/m, and then to study the impacts of changing electric field strength, the experiment was repeated at 500 and 2000 V/m. Time lapse images from these runs are shown in Figure 4.

For this surfactant formulation (solution 4), RB visibility is greatly reduced, but the motion of fluorescein dye is clearly visible. By comparing results for solution 4 with that of solutions 1–3 (solutions containing only one surfactant) at 1000 V/m, it can be observed that the separation pattern appears similar, with fluorescein migrating to the anode first and then eventually being contained within the bottom half of the device. After 10 min of operating under 1000 V/m, a fluorescein exclusion zone (RB band) that occupies 30% of the width of the device has formed, comparable to the performance of solution 1. Figure 4 shows that increasing the electric field strength increases the speed of dye migration and separation. The channel width of the fluorescein exclusion zone at 10 min increases from 23% for 500 V/m to 30% at 1000 V/m and to 44% at 2000 V/m. The color intensity of the fluorescein fronts increases with the electric field strength as the pH gradient forms quicker due to the intensity of electrochemical reactions induced by the higher electric fields. However, electric field strength has impacted the foam stability negatively. At 15 min, foam collapse at 1000 V/m appears more advanced than at 500 V/m and at 2000 V/m, the foam has almost entirely collapsed. Foam collapse between 15 and 20 min for 500 V/m appears relatively minor, while significant decay is observed at 1000 V/m. At 2000 V/m, the foam is completely collapsed within 20 min. This result suggests that the external electric field strength can be varied to achieve faster separation and tailor the width of the resulting analyte bands. Furthermore, once the required separation has been achieved within the separation chamber and the foam has entered the outlet channels, a high external electric field at the outlets can be used to collapse the foams for ease of collection or further processing. The effects of varying the initial dye concentration on separation were probed by doubling and halving the original dye concentration used with solution 4. No noticeable change in separation pattern was observed, and the results are presented in the Supporting Information (Figure S10).

### Effect of Initial pH on Separation

**Figure 5:**
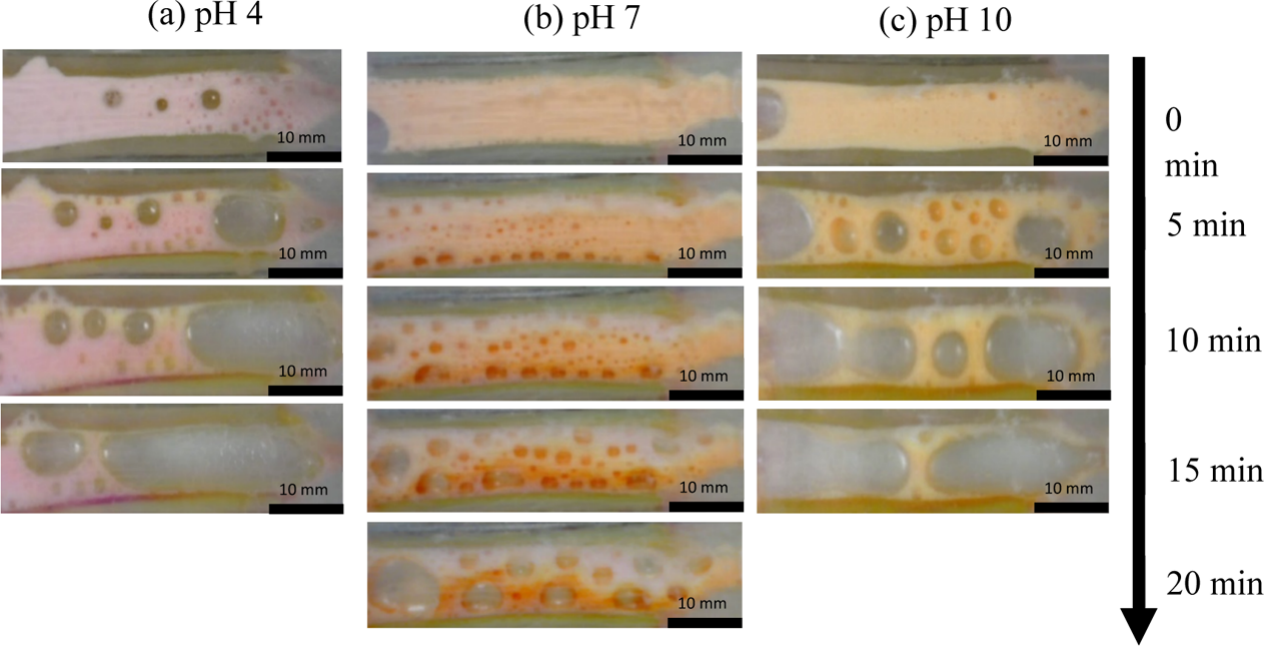
Time lapse images for the SDS/Triton X-100 blend at 1000 V/m, (a) pH 4, (b) pH 7, and (c) pH 10. The cathode is situated at the top of the edge of each image and the anode at the bottom.

To investigate the effect of changing the initial pH of the system, two more experiments were run using SDS/Triton X-100 mixtures. Solution 5 was prepared with phthalate buffer solution (pH = 4), while solution 6 was prepared with borate buffer solutions (pH = 10). Both solutions were run at 1000 V/m in the device. Time lapse images from these experimental runs are shown in Figure 5, alongside a run at pH 7.

As shown in Figure 5a, the dye mixture initially appears pink for an initial pH value of 4 as the acidic conditions increase rhodamine visibility while greatly reducing fluorescein visibility. At this pH, fluorescein is expected to exist in its neutral form throughout the foam. When an external electric field is applied, a faded yellow band appears at the cathode after ∼5 min as the pH increases due to electrochemical reactions at the cathode, causing fluorescein visibility to increase. The pH 4 case was repeated by replacing the dye mixture with methyl violet to check whether the pH drops below 2 near the electrodes. No color transition was observed (Supporting Information, Figure S5). This indicates that the solution pH remains above 2 during the experiments, and the cationic form of fluorescein dye is not present in these experiments. The fluorescein appearance near the cathode is due to local pH changes rather than electrophoretic transport. In all other cases examined, fluorescein was removed from this region upon the application of an electric field. After 10 and 15 min, this yellow band has grown slightly, acting similarly to the darker orange fluorescein bands observed in Figures 2a,c and 4, but in the opposite direction, extending from the cathode instead of the anode. Foam stability appears greatly reduced during this experiment possibly caused by the replacement buffer. A clear separation is not achieved at this pH.

When the initial solution pH is set to 10 using boric buffer, as shown in Figure 5c, rhodamine visibility is reduced to the point where rhodamine appears colorless (see Supporting Information, Figure S8). The starting dye appears as a brighter orange compared to the cases at pH 4 and pH 7. After an external electric field is applied for 5 min, a narrow white band starts to appear at the cathode, when anionic fluorescein is transported away. In contrast to the cases with neutral starting pH, no dark orange band forms in the foam at the anode, and the fluorescein absorbs into the agar gel instead. In this case, the pH within the foam is unlikely to drop below 4.3 during most of the experiment as the starting buffer pH is 10 (see Supporting Information, Figure S4). Under these conditions, fluorescein maintains its anionic form. After 10 min, the lighter yellow band seems to widen as more fluorescein continues to concentrate at the anode. Similar to Figure 5a, foam stability is greatly reduced by the replacement buffer, causing premature collapse compared to the other cases. This may be caused by the change in the zeta potential for the higher pH case, resulting in higher EOF and leading to reduced stability.

Adjustment of the starting pH may allow the system to be tailored to achieve different goals. For example, if the objective were to recover pure rhodamine in this case, a starting pH of 10 may be preferred as the fluorescein will be drawn to the anode for most of the operation, ultimately concentrating in a small region by the anode or even contained within the agar gel. If the objective were to recover fluorescein, then a starting pH of 7 may be ideal. In this case, fluorescein will concentrate in the bottom half of the device rather than absorbing into the agar where recovery may become difficult. Separation may potentially be enhanced by recycling fractional output back through the device, and the possibility of running the system continuously will be an interesting topic for further research.

## Conclusions

The three-dimensional foam electrokinetic experiments presented in this paper demonstrate the potential of liquid foams as a separation platform for charged molecules, specifically a mixture of dyes. In addition to the molecular transport by electrophoresis and electroosmosis expected in a typical microchannel under an external electric field, interaction of charged species with the surfactant laden gas–liquid interface charge becomes important in foam separations. The type of surfactant or the surfactant mixture used to generate the foam has been shown to affect the separation significantly by not only affecting the magnitude and the direction of EOF inside the foam system but also the positioning of charged molecules closer to or away from the gas–liquid interfaces. Foams produced with non-ionic surfactants have shown better separation compared to foams made with charged surfactants due to suppressed EOF and improved foam stability under external electric fields. Within the experimental range of 500–2000 V/m, stronger electric field strengths have shown to accelerate the separation but lead to rapid destabilization of foam, which can limit the maximum electric field strength for specific applications. As the lifetime of a foam is limited under external electric fields, the final lateral position of analytes may also be controlled by altering the initial pH of the system. pH could change the charge status of molecules or particle zeta potential; therefore, selecting an appropriate initial solution pH using buffers could be beneficial for a given separation system. The foam electrokinetic system demonstrated here may enable development of novel separation techniques for mixtures that are hard to separate, such as mixtures of small proteins and ions. Further work is required to develop a continuous separation system with foam flow and product separation.

## Supporting Information Available

Chemical structures of surfactants trialled; time lapse images of pH changes inside foam using pH indicators; initial colors of unmixed dyes for each surfactant trialled and at different pH; foam containing a single dye under external electric fields; and effect of the dye concentration on foam electrokinetic separation (PDF)
