# Supplementary material for: Foam-Based Electrophoretic Separation of Charged Dyes
Source: Langmuir. 2022 Nov 2;38(45):13935–42. doi: 10.1021/acs.langmuir.2c02228 (PMC9671044; doi:10.1021/acs.langmuir.2c02228)
Supplement: Supplementary file 1 — la2c02228_si_001.pdf [file la2c02228_si_001.pdf]

Supporting information

Foam based electrophoretic separation of charged dyes

Matthieu Fauvel [a], Anna Trybala [a] Dmitri Tseluiko [b], Victor Mikhilovich Starov [a], Himiyage Chaminda Hemaka Bandulasena[*a]

[a] Department of Chemical Engineering, Loughborough University, Loughborough, Leicestershire, LE11 3TU, United Kingdom

[b] Department of Mathematics, Loughborough University, Loughborough, Leicestershire, LE11 3TU, United Kingdom

[*]**Correspondence:** H.C.H.Bandulasena@lboro.ac.uk; Tel.: +44-1509-222515

Surfactant chemical structures

Chemical structures of Sodium Dodecyl Sulphate (SDS), Myristyltrimethylammonium bromide (MTAB) and Triton X-100

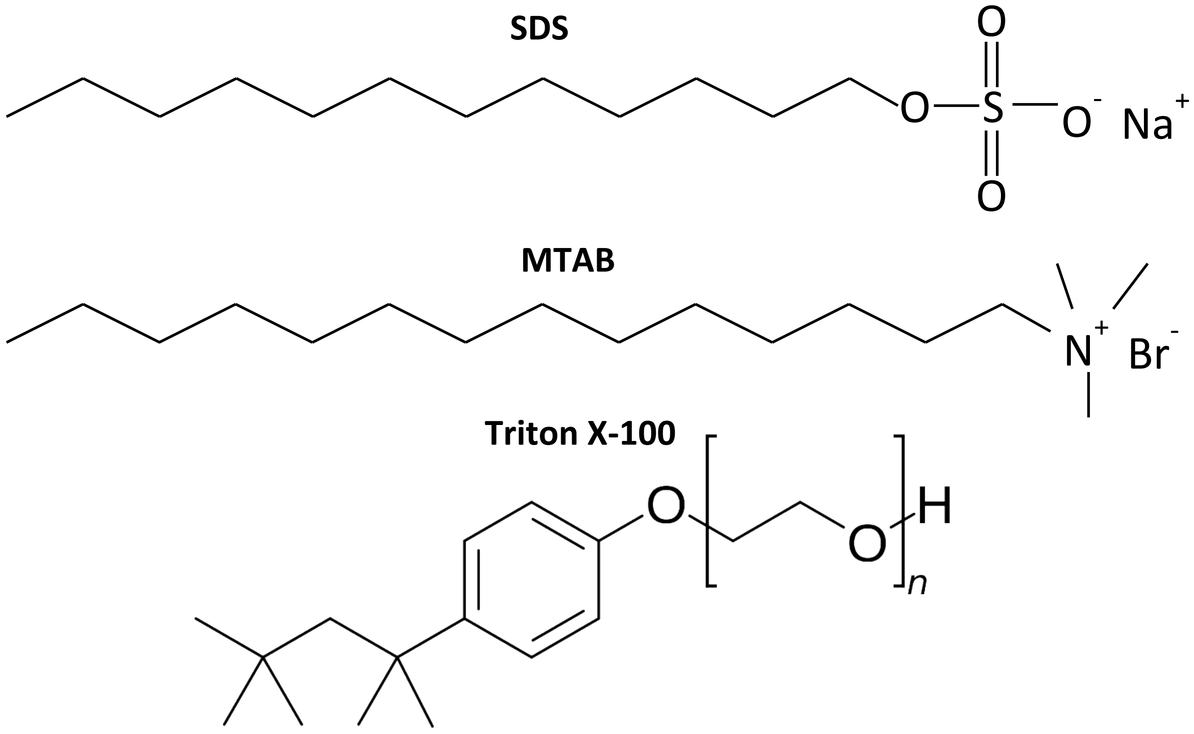

Figure S1: Chemical structures of SDS, MTAB, Triton X-100

pH changes in foam under an external electric field

A universal indicator is added to solution 4 (containing both SDS and Triton X-100 at 1 CMC) in place of rhodamine and fluorescein, and the device was operated with 1000 V/m. Time lapse images from the run are displayed in Figure S2. In these images, green, yellow and blue regions correspond to approximate pH of 7, 4 and 10 respectively. Colour changes for solutions 1, 2 and 3 (containing either SDS, MTAB or Triton X-100) looked similar.

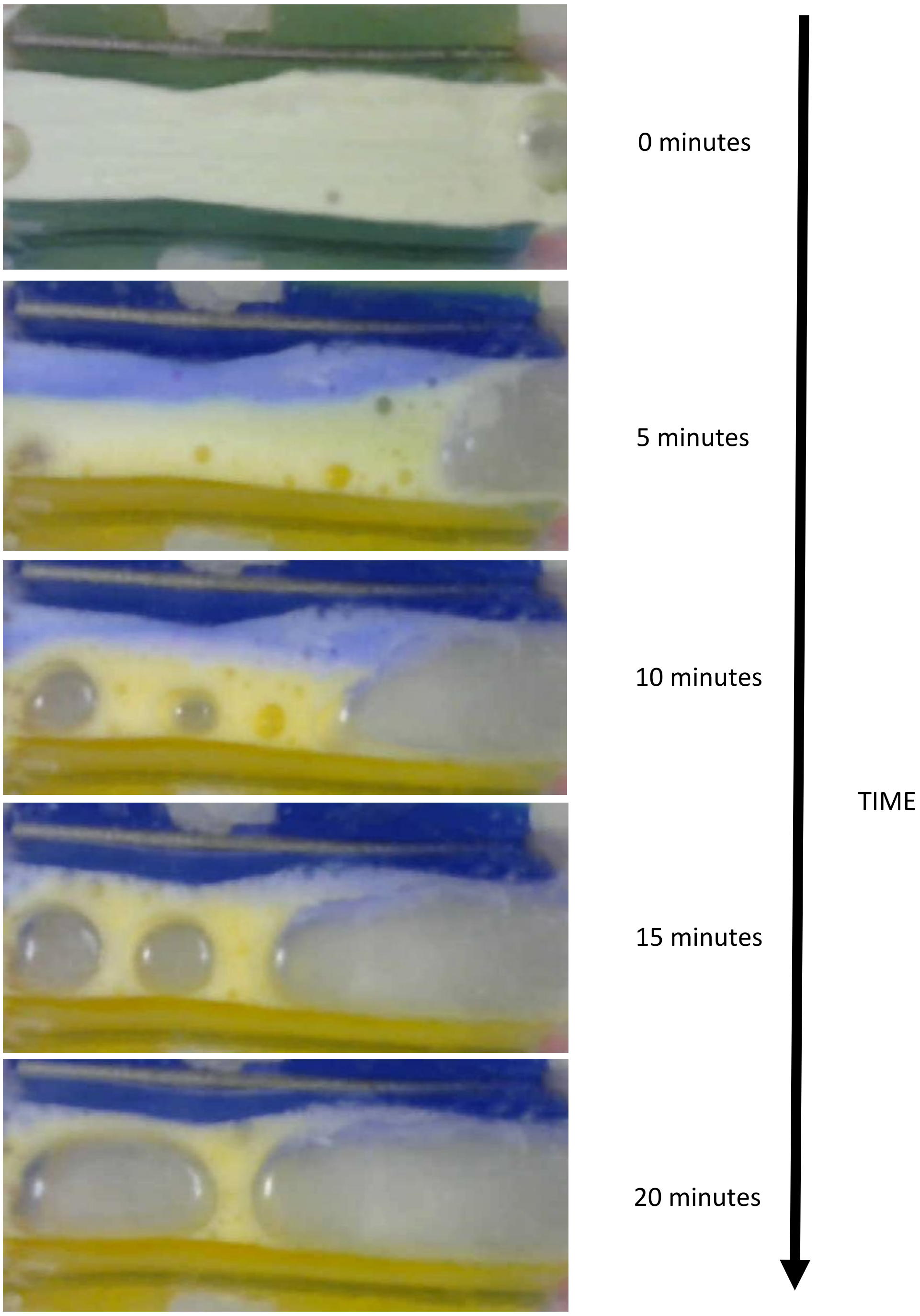

Figure S2: pH changes in foam prepared with a mixture of Triton X-100 and SDS at 1000 V/m at a starting pH of 7.

Universal indicator is added to solution 5 (phosphate buffer replaced with phthalate buffer) in place of rhodamine and fluorescein, and the experiment is repeated. Time lapse images are displayed in Figure S3.

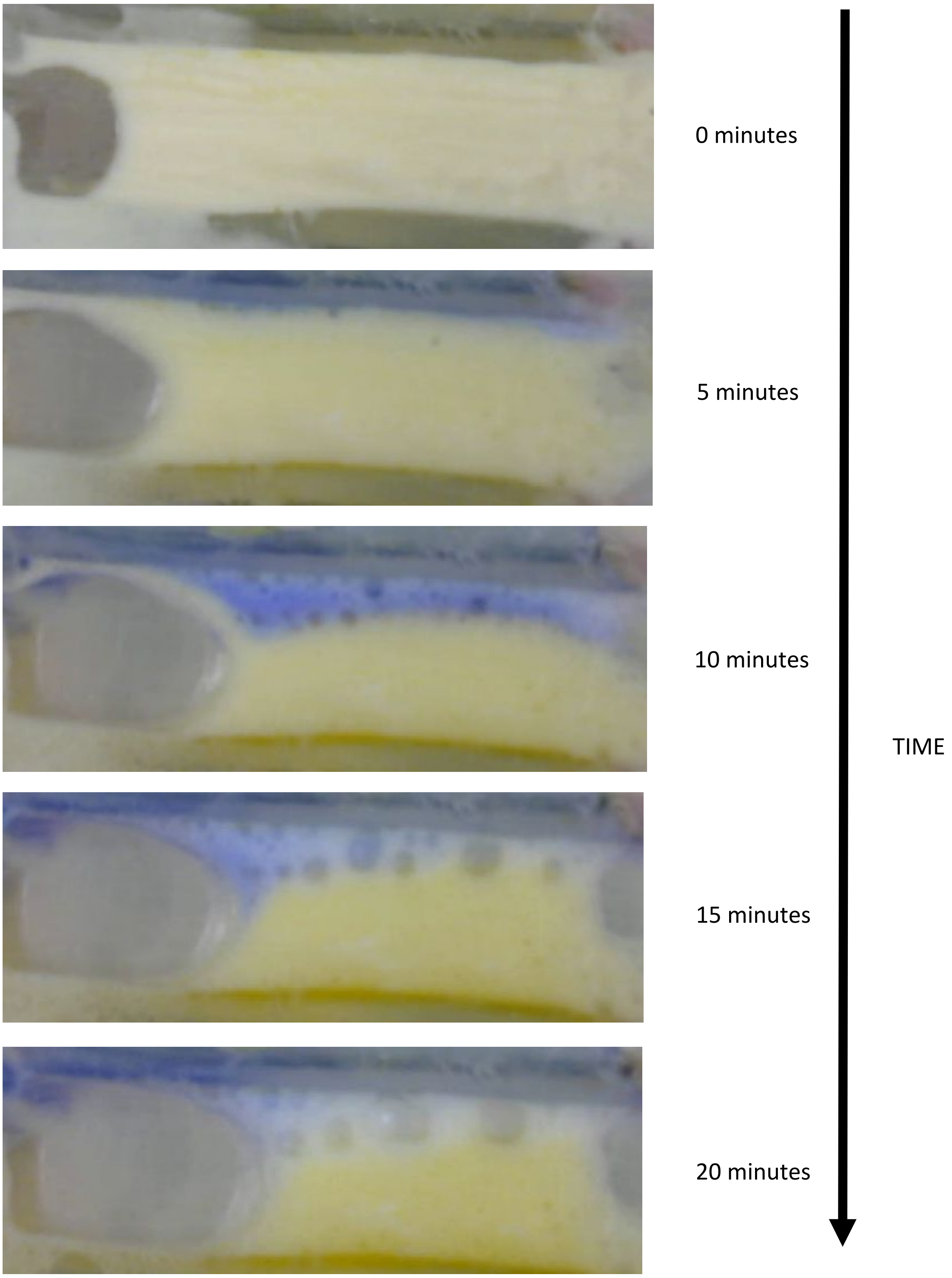

Figure S3: pH changes in foam prepared with a mixture of Triton X-100 and SDS at 1000 V/m at a starting pH of 4.

Universal indicator is added to solution 6 (phosphate buffer replaced with borate buffer) in place of rhodamine and fluorescein, and the experiment is repeated. Time lapse images are displayed in Figure S4.

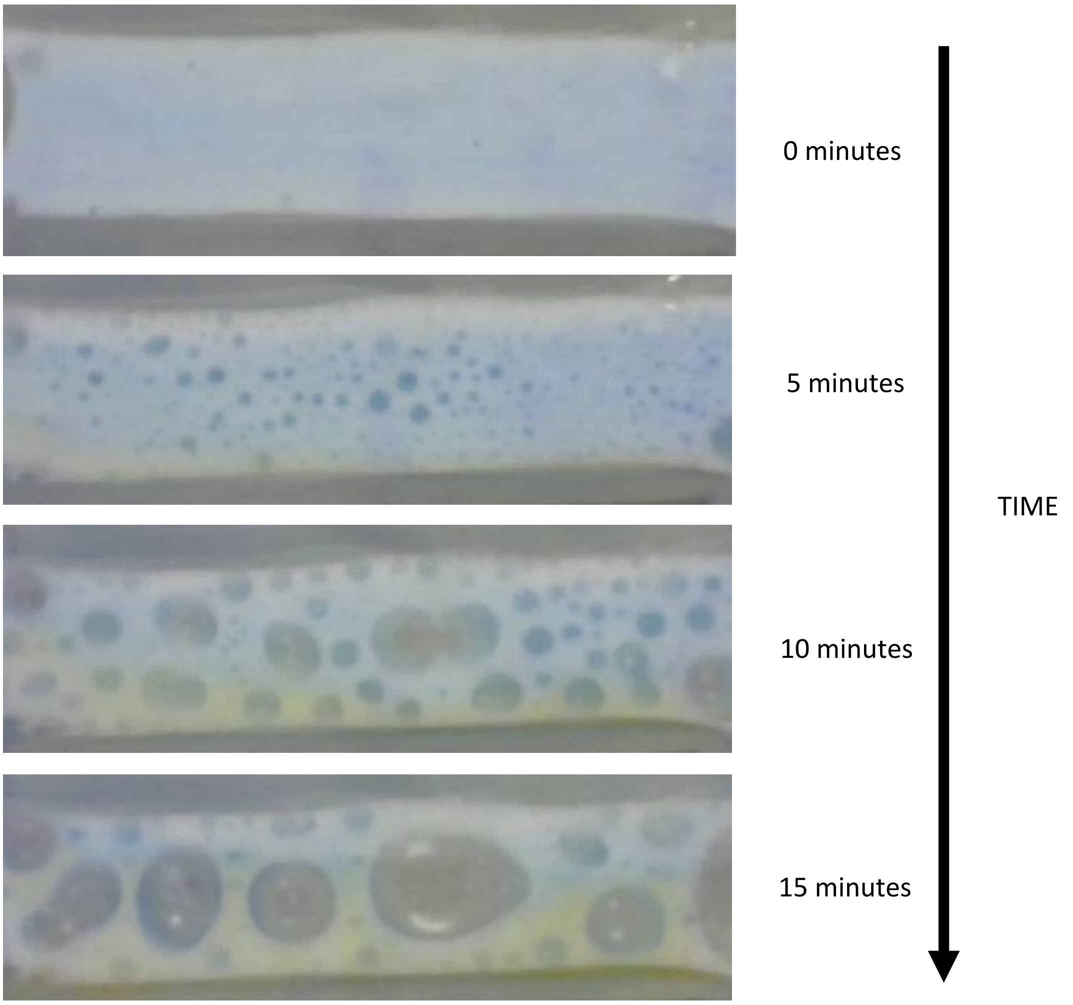

Figure S4: pH changes in foam prepared with a mixture of Triton X-100 and SDS at 1000 V/m at a starting pH of 10.

Methyl Violet is added to solutions 4, 5 and 6 in place of rhodamine and fluorescein. The experiment is run at 1000 V/m for these three solutions. Time lapse images are displayed in Figure S5.

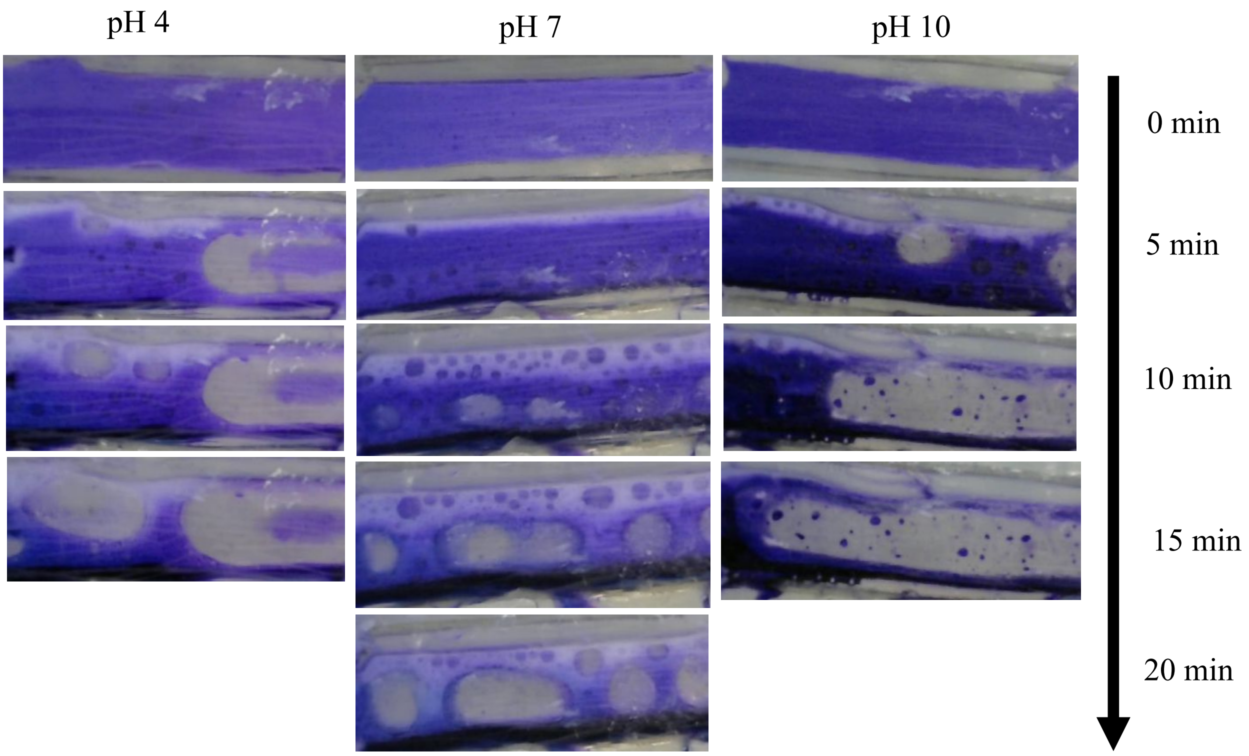

Figure S5: Time lapse images for methyl violet in SDS/Triton X-100 blend at 1000 V/m, pH 4 (left), pH 7 (centre), pH 10 (right).

Initial foam colours with pure dyes

The colours of unmixed rhodamine B and fluorescein in each surfactant solution are displayed in Figure S6.

Rhodamine | **Fluorescein**

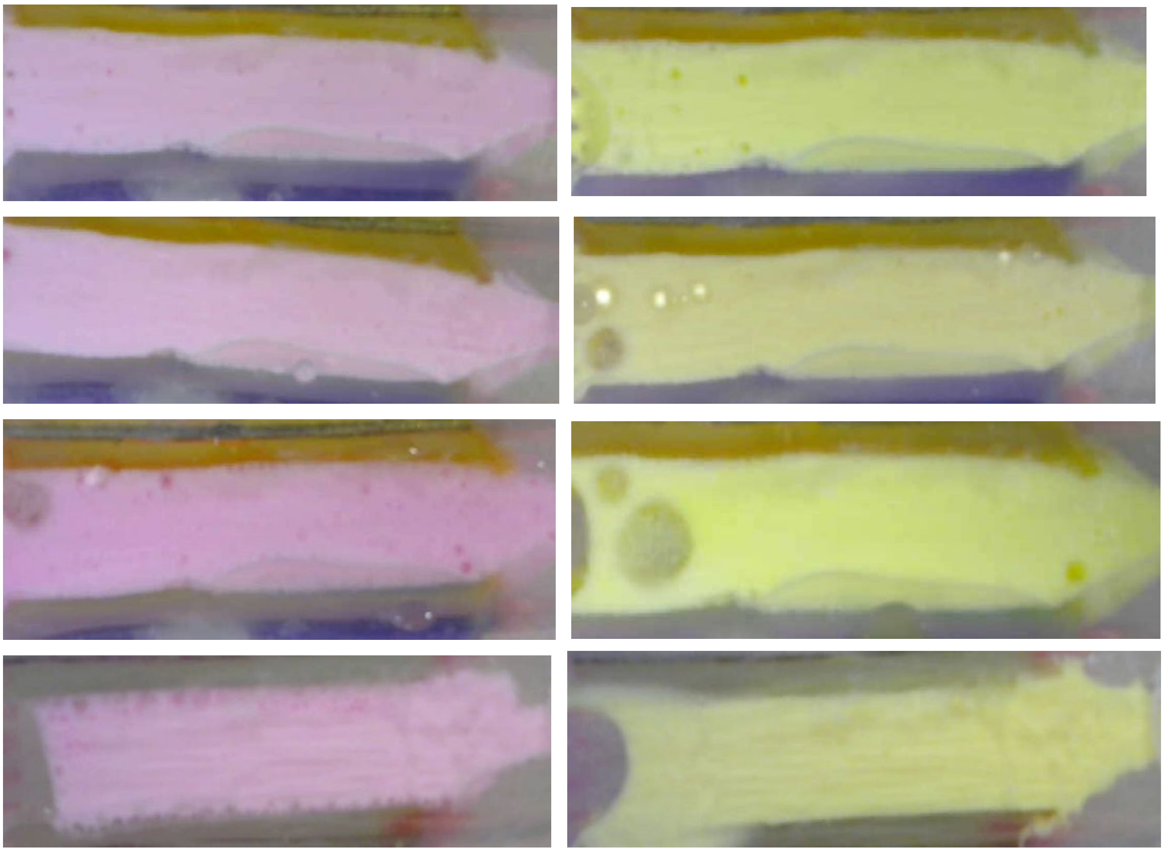

Figure S6: Initial colours of Rhodamine B and Fluorescein in each solution. In order from top to bottom: SDS, MTAB, Triton X-100, SDS/Triton X-100 mixture

The initial colours of each dye at varying pH levels is shown in Figures S7 and S8:

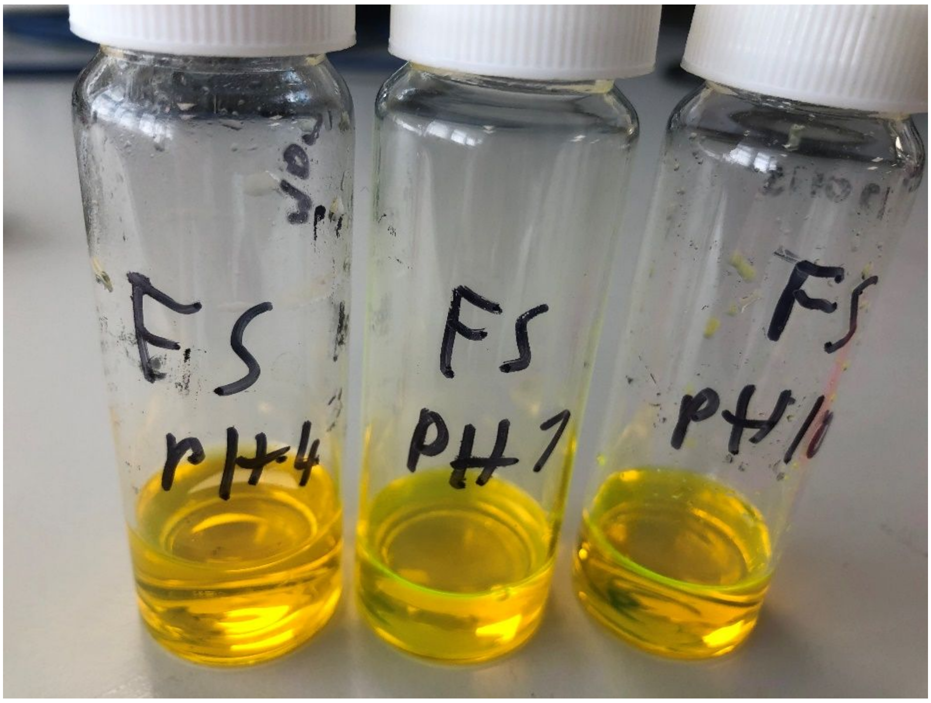

Figure S7: Fluorescein solution at pH 4 (left), pH 7 (centre) and pH 10 (right)

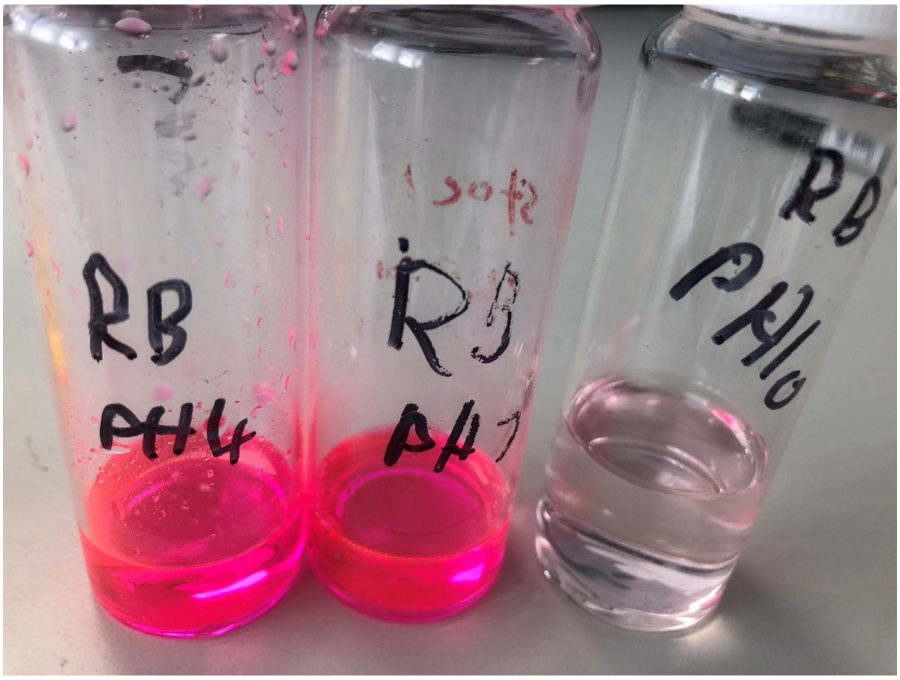

Figure S8: Rhodamine B solution at pH 4 (left), pH 7 (centre) and pH 10 (right)

Time-lapse images of unmixed rhodamine B and fluorescein SDS solution under applied electric field of 1000 V/m is displayed in Figure S9.

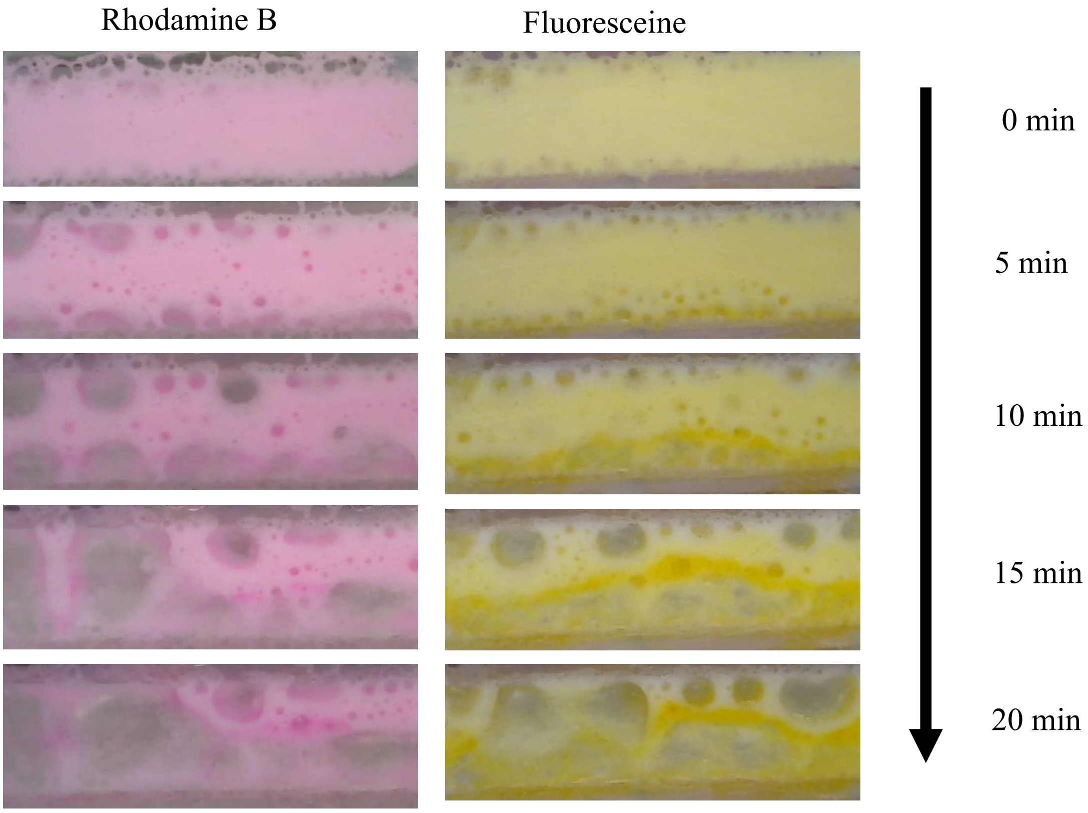

Figure S9: Time lapse images for Rhodamine B and Fluoresceine in SDS solution at 1000 V/m.

To assess the effect of varying dye concentration on separation, two variations of solution 4 were made, one with half the original dye concentration (Fluorescein and Rhodamine B concentrations of 0.25 mM and 0.05 mM), and one with double the dye concentration (Fluorescein and Rhodamine B concentrations of 1 mM and 0.2 mM). Time lapse images of these solutions under a 1000 V/m electric field are displayed in Figure S10.

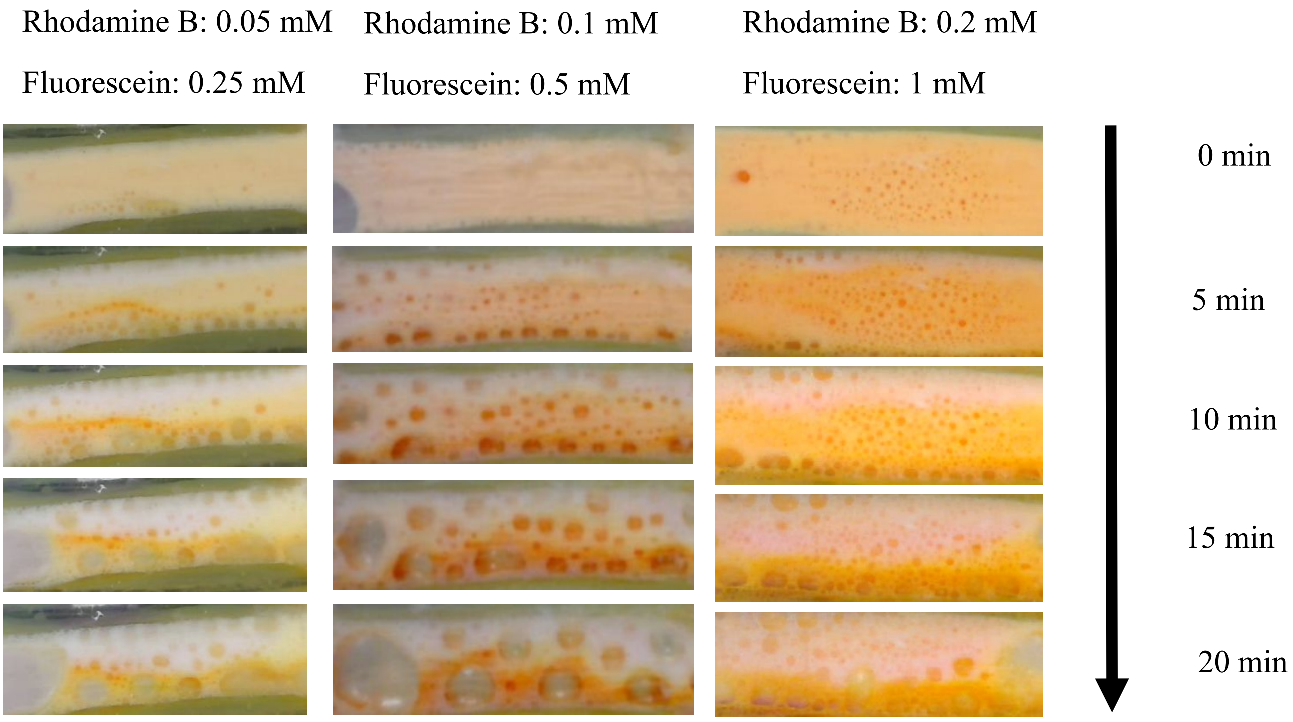

Figure S10: Time lapse images of varying dye concentrations of Fluorescein and Rhodamine B in SDS/Triton X-100 mixture at under 1000 V/m.
